# Maintaining and Supporting Seniors’ Wellbeing through Foreign Language Learning: Psycholinguistics of Second Language Acquisition in Older Age

**DOI:** 10.3390/ijerph17218038

**Published:** 2020-10-31

**Authors:** Marcel Pikhart, Blanka Klimova

**Affiliations:** Department of Applied Linguistics, Faculty of Informatics and Management, University of Hradec Kralove, Hradec Kralove 500 03, Czech Republic; blanka.klimova@uhk.cz

**Keywords:** positive psychology, foreign language learning, FLL, psycholinguistics, psychology of education, education of seniors, wellbeing, healthy lifestyle improvement, mental health, brain health, L2 acquisition, second language acquisition

## Abstract

This study concerns aspects of positive psychology connected to foreign language learning (FLL) in an older healthy generation. The positive psychology perspective stresses the positive aspects of improved wellbeing in participants who engage in various activities, particularly mental and brain-training practices. Therefore, the aim of this study was to explore older people’s subjective feelings connected to their FLL as one of the crucial ways to improve their quality of life (QoL). The objective of the research was to determine the subjective satisfaction level of the participants of a second language (L2) acquisition course. The research sample (experimental group) consisted of 105 respondents who were Czech citizens and 55+ years old. Two control groups were set up. The first (young control) consisted of 102 young adults (university students), also Czech citizens, aged between 19 and 23 years. The second control group (elderly control) consisted of 102 subjects older than 55 years, similar in age to the experimental group. A standardized online questionnaire survey was the principal research method, identical both for the experimental and control groups. The findings clearly showed that language training significantly improved the subjective positive feelings and wellbeing of the older participants, regardless of their objective progress in FLL itself. These results stood in opposition to the young control group and were different from the elderly control group. The results revealed that FLL is an effective tool for enhancing the overall wellbeing of older people, which was shown in their expression of their feelings of happiness, satisfaction, and positive motivation to learn an L2. In addition, FLL objectively affected their mental health in a positive way and expanded their social networks. Moreover, FLL was a meaningful activity for them, despite the weak objective learning outcomes due to the decline of cognitive functions, helping them find their general purpose of life, as well as life motivation as expressed in the survey. These findings are crucial, as it has already been proven that wellbeing is directly connected with good health and longevity. Therefore, national governments and all stakeholders dealing with the present issue of the aging population should pay undivided attention to the enhancement of older people’s wellbeing by all possible intervention approaches, including FLL. There is limited research into the issue and the findings of this investigation could be an impetus for further research into the topic from the perspectives of cognitive science, psychology, and psycholinguistics.

## 1. Introduction

Currently, due to demographic changes, there is an increase in the number of older generation groups, which results in serious social and economic issues, especially in the rising costs of care for these older people. Moreover, there is an effort to prolong the active life of these people, as well as to maintain and/or enhance their quality of their life (QoL) through their improved subjective wellbeing [1]. Wellbeing can be defined as an individual’s subjective experience of health, happiness, and prosperity, which is reflected in good mental health, subjective high life satisfaction, a sense of meaning or purpose of life, and ability to reduce or manage stress efficiently [2]. The improvement of one’s wellbeing can be done in different ways [3], and we claim foreign language learning (FLL), or second language (L2) acquisition, to be one of them. This paper attempts to analyze how much FLL or L2 acquisition in older age can help to improve subjective QoL and increase feelings of happiness. 

FLL may play an important role in the lives of healthy older people (i.e., aging normally with no serious pathologies) since acquiring new knowledge and skills is possible at any age [4]. Recent research indicates that the brain retains its plasticity even in older people [5], and almost any cognitive training can stimulate and support lifelong cognitive plasticity [6]. An example of such training might be a short, but intensive, foreign language course, which can lead to a significant improvement in general cognition with increased functional connectivity [7].

Research studies show that FLL can enhance both cognitive [8,9,10,11,12,13,14] and non-cognitive [15,16,17,18,19,20] functions in seniors, thus contributing to their wellbeing and, consequently, to their improved QoL. Cognitive functions are mainly associated with the mental state, which is affected by information input, processing, and transfer, while non-cognitive functions are mainly connected with attitudes, beliefs, and attributes [21]. However, both these dichotomic components are inseparably intertwined and are crucial for the individual’s developmental process at any age [18], being deeply rooted in the individual overall approach to life. The non-cognitive aspects of FLL are especially visible among seniors whose motivation for FLL is considerably influenced by, first, their social wellbeing [22], i.e., the ability to communicate, establish meaningful relationships with others, and develop a support network of new friends in order to reduce loneliness, and second, by emotional wellbeing [23], i.e., the ability to manage stress and develop positive emotions. Both these stimulations for FLL are essential at a senior’s age and positively influence their language achievements. Pfenninger and Singleton [24] state that FLL in older age must be considered not just as an aim in itself but as a means of developing social inclusiveness and networking, which is partly done through the stimulation of social wellbeing where its cognitive effects may, potentially, be observed. This point of view is especially supported by positive psychology theory aimed at improving learners´ linguistic competence through enhancing learner experience of fun, hope, courage, optimism, creativity, happiness, or resilience [19]. As described above, apart from the interest in language, it is mainly a social stimulus that motivates people to learn a new foreign language. However, ideally, they must be in the group of learners of the same or similar age, i.e., in the group of seniors. Otherwise, they might feel uncomfortable and anxious because younger adults are much better in their retention and faster in their reaction, and therefore more impatient when older adults want to reply in an elaborative and precise way [25]. 

As far as the research is concerned, there are many studies on FLL with respect to positive psychology conducted among young learners (see [20]); however, there is a lack of research studies on older generation groups and their motivation to learn a foreign language as well as the impact it can have on their subjective feelings of satisfaction and wellbeing, and, subsequently, their QoL [24]. Therefore, the aim of this study was to explore older people’s subjective feelings connected to learning a foreign language as one of the ways for improving their QoL as a way of active aging [26] or active social aging [27]. QoL of seniors can be improved in many ways, such as conducting physical activities [28], following healthy nutrition [29], performing music [30], being involved in a social environment [31], and various social activities whose aims are to improve cognitive skills. FLL is one of them, but it is rather neglected despite its great potential, as this study reveals. 

## 2. Materials and Methods

This paper attempted to evaluate the level of subjective satisfaction of the respondents with the language course they attended and claimed that the older respondents from the experimental group would reach higher levels of satisfaction than in the control group of younger learners (young control) and the control group of seniors (elderly control) who did not engage in FLL activity. All the respondents in the experimental group and young control group were on a very similar level in terms of their FLL, namely, pre-intermediate and intermediate, i.e., A2 or B1 according to the standardized Common European Framework of Reference for Languages (CEFR).

Naturally, all the participants might have had different motivations in terms of taking part in the course, but was is not possible at all to find all the people with identical motivations to learn a foreign language. We tried to compare the groups of leaners who studied the L2 for their intrinsic motivation. In order to have comparable data with the experimental group, we did not include any university students in the control group who studied the L2 as their major or minor. We also believe that the large sample in all three groups enabled us to draw solid conclusions regarding the enhancement of their wellbeing. In the elderly control group, we had participants who did not study a foreign language and, on the contrary, in the young control group, we had a majority of students who studied a foreign language only from their internal motivation and pleasure, not for assessments or objective results (mostly students from Chinese classes). For these reasons, we claim that their motivations to study a language for pleasure or to pursue some other interesting activity, such a hobby (in the experimental, young control, and elderly control groups), was nearly the same. 

### 2.1. Research Sample

Within this paper, people over 55 years old were considered to be seniors. Although this may seem counterintuitive, as they mostly are active and working persons, their cognitive (learning) operations require approaches specific to this age group, i.e., adult learning [32]. In addition, the starting age to retire in the Czech Republic is 55 years of age [33]. 

**Figure 1:**
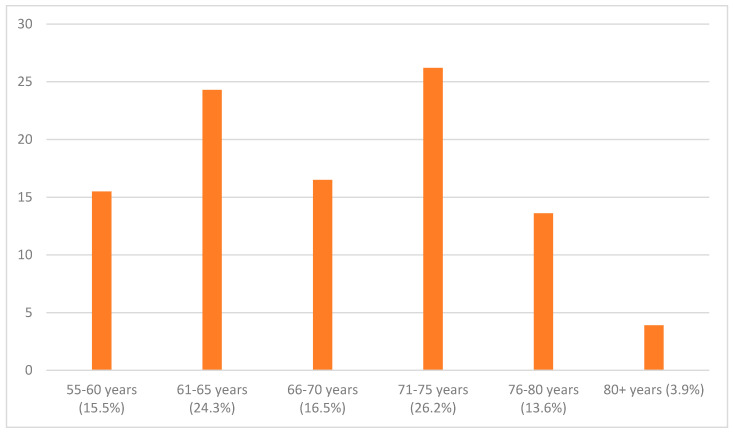
Age distribution of the experimental group.

Thus, the research sample of participants consisted of respondents between 55 and 80+, belonging to the six distinct age-groups shown in Figure 1 (the brackets indicate the percentage of respondents). The largest group was the segment of 71–75 years (with 26.2%), and the second largest was the segment of 61–65 years (24.3%), together making the total of more than a half of all the respondents.

The research sample consisted of 105 respondents (*n* = 105) who were 55 years old or older. The upper limit was not set, and the oldest respondent was slightly over 80 years. The age distribution of the respondents was evenly balanced, however, the segment of those aged 80+ years was very small for obvious reasons. Age was relevant only as a reference point, i.e., the only crucial point was if the respondent was older than 55 because the paper aimed to look for the intrinsic subjective feelings and positive psychological outcomes of the L2 acquisition in the older generation, i.e., people older than 55 years. The research did not focus on any age segmentation in L2 retention connected to age.

All of the respondents were Czech citizens, with some of them studying a foreign language at the University of Hradec Kralove, the Czech Republic. All of these respondents were students enrolled in the program of the University of the Third Age (generally known and commonly abbreviated as U3A). The idea behind U3A is to provide seniors with certain courses that could help them to improve their knowledge, skills, and competencies (in this case, the L2 acquisition). The second group of the subject sample (all of them were Czech citizens) consisted of active seniors who still had a full-time or a part-time job in a company or those who took part in language courses organized as company courses. It was not statistically relevant to divide the participants into two groups, i.e., U3A or company courses. The most important criteria was the fact that these older people took part in language courses and that they were older than 55 years, with the research again focused on their subjective feelings about their L2 acquisition. All the respondents in this group were self-enrolled in the courses. All the respondents proved to be very active seniors (either because of their enrolment in the U3A or their current job) with personal and intrinsic motivation to take part in the language course. All the courses were on a very similar level, i.e., A2 or B1 according to CEFR, and all of them had the same time range, i.e., 90 min per week for 13 weeks in the winter and 13 weeks in the summer semester. There was no significant attrition in the number of the participants during the course, usually only a few participants. 

The data were collected anonymously and neither IP addresses nor names or any personal information was collected. The only individual identification of the questionnaire is the time stamp connected to the questionnaire itself and not to the personality identification of the respondent. The general European Union GDPR regulation (General Data Protection Regulation, www.gdpr.eu) was fully followed and there were no issues regarding privacy according to the Czech and European law. All the respondents were informed about the research and they explicitly expressed their agreement with being involved in the research activity. 

There were two control groups, i.e., the young control and elderly control. The young control group for this research consisted of young adults studying at the University of Hradec Kralove and University of Pardubice, Czech Republic, and attending various language courses provided by the universities (i.e., they were not studying languages as their major or minor). They studied at the same university as the experimental group and they attended very similar language courses, including those conducted by the same tutors. The students were self-enrolled in some of the courses, and some of the courses were not compulsory for them (e.g., Chinese). For this reason, they may have had different motives for learning a language compared to the experimental group. Their age was between 19 and 23 years. The same questionnaire was sent to them with the same methodology and parameters to obtain results to be compared with the experimental group. This sample consisted of 102 respondents (*n* = 102), which was almost the same number as the experimental group for comparison purposes. 

**Figure 2:**
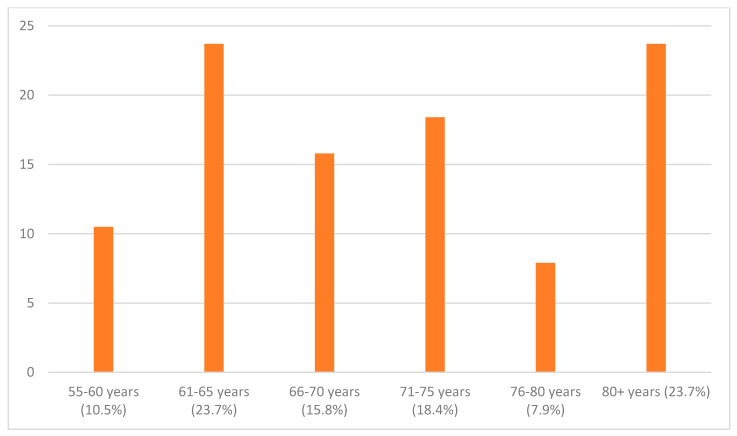
Age distribution of the elderly control population.

The second control group (i.e., elderly control) consisted of seniors (*n* = 102) who did not attend any organized language course but had other free time activities. The exclusion criterion for this was any language course they would be attending at the time of the study. The research sample consisted of 102 respondents from the same region and of very similar age and education structure. Similarly, the second control group consisted of respondents between 55 and 80+. They belonged to the six groups shown in Figure 2 according to their age (the brackets indicate the percentage of respondents), and they more or less corresponded to the experimental group, with a higher number of respondents in the last group, i.e., 80+. The largest groups of the respondents were the age groups of 61–65 (23.7%) and 81+ (23.7%), encompassing nearly 50% of all the respondents. 

### 2.2. Research Methods

The principal research method was an online questionnaire survey that was modified according to a standardized questionnaire by Woll and Wei [34] by selecting key questions connected to the subjective feelings of satisfaction and adding a few filler questions from the same standardized questionnaire. The modified questionnaire focused on the positive feelings of the respondents, which could be associated with any L2 acquisition in any context with the aim of collecting basic data about the subjective satisfaction. The individual items for the questionnaire were carefully selected from the given questionnaire [34] in order to obtain as much information as possible in a limited set of statements (total 20 key items). The questionnaire was designed so that it would not take up more than 10 min of the respondents’ attention to minimize the attention span of the experimental group and the elderly control group. 

Following a short introduction enquiring about the respondent’s age, L2, place of residence, and education, the questionnaire only contained 20 statements, some of which focused on foreign language learning from a positive psychology perspective. There were a few filler questions focusing on travelling, getting to know other cultures, and how much free time is devoted to FLL. The last point of the questionnaire was an open question where the respondents could express their opinions, feelings and ideas connected to the language courses. This was considered to be a filler question as well; however, it generated some helpful insights that could be qualitatively analyzed while looking for the respondents´ motivation and other important aspects of their participation. These ideas added a certain value when evaluating the quantitative data obtained by the questionnaire. 

The questionnaire was distributed online through Google Forms as a very useful tool to obtain the data from the respondents quickly and reliably. The respondents were asked to fill in the form anonymously and submit it to the researchers. The respondents were contacted by an email link only or informed personally about the research, followed up by an email link to the form of the questionnaire. Overall, 100% of the contacted potential respondents reacted and replied due to the fact that the questionnaire was very intuitive and short. The data collection period took place in April and May 2020. The respondents replied almost immediately after an email was sent to them or after being contacted in person by the tutor of the course with a follow-up email. 

The research statements focused predominantly on the responses of positive feelings associated with foreign language learning, including several filler questions (see questions 6, 7, 8, and 9, for example) connected to travelling and meeting friends, etc., and thus the respondents did not know that the questionnaire was focused on their subjective wellbeing and feelings. Moreover, the title of the questionnaire indicated that it concentrated on their satisfaction with the language courses and the tutors. The filler questions were also directed this way. In reality, the questionnaire neither focused on their satisfaction with the course, nor the objectively measured results of their language progress, nor the general satisfaction with the tutor. The only focus of the research was on their intrinsic positive feelings that could be associated with foreign language learning. The idea of this research was not explicitly formulated anywhere in the questionnaire in order to avoid respondents´ bias regarding the scope and the focus of the research. On the contrary, the introduction to the questionnaire stated that the research focused on their satisfaction with the course itself. 

A 6-point Likert scale was used in the questionnaire, offering options from

- Strongly agree;
- Agree;
- Agree a little;
- Disagree a little;
- Disagree;
- Strongly disagree.

Twenty statements were used to test and analyze the respondents´ perception of their L2 acquisition from the perspective of positive psychology, regardless of their objectively measured results in their L2 acquisition, i.e., their objective learning outcomes were not relevant for the study whatsoever. The individual items of the questionnaire are cited in Table 1.

It was important for the research that the respondents were not supposed to know that the research focused on the positive psychology aspects of L2 acquisition, but the researchers claimed, at the beginning of the questionnaire, that the survey was conducted to improve the general quality of the courses.

## 3. Results

These results refer to the first part of the questionnaire related to the personal information questions. The experimental group consisted of 65% women and 35% men. Gender balance was not a relevant factor for the research result. Specifically, regarding the education of the respondents, as many as 58.3% of them had a university degree, only 1% (i.e., only one respondent) had only basic school education, and the remaining respondents had a high school degree. The vast number of respondents were either university-educated or high school graduates. Therefore, it was expected that these two groups would be more interested in further learning even in their older age. Their increased interest in further education could also be the reason why they took part in U3A. 

The respondents were also divided according to their permanent address, i.e., whether they lived in a city/town or a village. A total of 70.9% of them were from towns or cities (more than 10,000 inhabitants), whereas 29.1% were from villages. This was supported by the idea that people in towns and cities have a more active life, as well as being due to increased learning and cultural opportunities (such as U3A courses). 

The job structure of the respondents was very wide, ranging from technical positions to teachers, doctors, nurses, accountants, building engineers, chemists, and hotel receptionists to a few factory manual workers. The majority of the respondents (58.3%), however, fell into the category of university-educated with corresponding jobs. Early life university education is likely to create a need for a further and life-long learning process, which explains the reason why more than half of the respondents had a university background. 

The L2 the respondents were studying was typically English or German. In the past, they would have mostly studied Russian, again for obvious historic reasons, because after the Second World War the Russian language was a compulsory language in the so-called Eastern Bloc, including Czechoslovakia (the current Czech Republic and Slovakia). Some of the respondents had previously studied other languages, such as Latin, Italian, Chinese, and French. The particular language studied at the time of this research did not have any impact on the feelings of wellbeing. 

The results showing the experimental group´s responses to the key statements are provided in Table 1 below.

The results of the research are very surprising when compared to the objective measures of learning outcomes. The students were tested after the semester regarding their acquired knowledge in vocabulary and grammar in their L2 using standard testing methods for L2 acquisition. The retention rate in L2 of the elderly students was very low, due to the natural aging process and the decline of cognitive functions, and it was very difficult for them to retain basic vocabulary and apply grammar rules correctly, as tested by current research into aging and cognitive decline. The natural aging process and cognitive function decline and all the related consequences connected to the L2 acquisition are described later in Section 4. The answers in Table 1 show a vast discrepancy between the objective L2 test results received (grammar and vocabulary acquisition tests after the course) when compared with the subjective satisfaction with the language learning process. The results of the tests were obtained by standardized test methodology provided by the textbook and they clearly complied with memory and cognitive decline progression that is well documented by research and literature (see Section 4). Again, on the basis of cognitive decline in older age connected to memory deterioration, the gap between the objective results and subjective satisfaction is counterintuitive when taking into consideration the very low objective results of the L2 acquisition of the learners. Even the students are aware that their progress in the L2 is very limited; however, their personal subjective satisfaction reached very high levels. Nevertheless, these results can be explained from a psycholinguistic and positive psychology perspective. 

To compare the results of the elderly control group, we submitted the same questionnaire with identical parameters (there was no difference in the methodology) to the young control group (i.e., the university students who are aged between 19 and 23), with the results summarized in Table 2.

Table 3 summarizes the results yielded from the elderly control group, i.e., the seniors who did not study any other language. The research focused on their hobbies and how much subjective feelings of happiness and wellbeing they bring. 

The results of the control group of the seniors who had traditional hobbies, excluding FLL (such as gardening, reading books, watching TV, sports, walking, family, cooking, pets), clearly showed that they enjoyed their hobbies very much and that they did not bring much stress, but the hobbies themselves did not create any significant improvement in wellbeing or their subjective QoL feelings when compared with the experimental group. The hobbies of this control group, naturally, were found to be extremely enjoyable as was visible from the results that were centered and balanced, but they did not tend to create extreme values in the areas of subjective happiness, unlike in the experimental group. The experimental group clearly showed extreme values in subjective happiness connected to the FLL, however, we could not find similar results in subjective happiness in elderly control connected to their hobbies. 

Regarding the first control group, i.e., the younger adults, the respondents did not show much improvement in their subjective feelings of happiness, despite their relatively good learning outcomes. From the traditional learning psychology perspective, which claims that good learning outcomes are a motivator, these results are not easy to fathom. On the contrary, low scores in FLL results are generally considered as a demotivator. In this research, there were other aspects that played a crucial role in the level of positive feelings connected to L2 acquisition of the respondents, and moreover, the yielded results were unexpected. 

When the results of the experimental group were divided into two halves, i.e., agree and disagree, regardless of the intensity of the agreement or disagreement, we obtained a clear-cut black and white scale. However, the most convincing results, i.e., strongly agree and agree, for learning a foreign language reflected their desire to travel and discover new countries; 92% of the respondents were positive about this and 88% also acknowledged the impact of FLL on discovering and understanding different cultures. In addition, over 80% of the respondents thought that learning a foreign language would enhance their cognitive skills, such as concentration and memory. The most important finding of this research was the idea that, regardless of whether FLL in older age can improve, retain, and maintain cognitive skills, it creates extremely high positive feeling levels connected to FLL. The same number of respondents (81%) also appreciated the social role of FLL manifested in finding new friends. This subjective appreciation of the enhancement of social bonds was one of the aspects that also brought about feelings of happiness and satisfaction. Furthermore, FLL was a positive motivation for 82% of the respondents in the experimental group, which was then reflected in their feelings of happiness and personal satisfaction. This subjectively experienced satisfaction, despite having low objective results in elderly people, is very important from a positive psychology perspective. Over 70% of the respondents also stated that FLL improved their creativity and helped them in learning other things. Again, it can enhance their improved wellbeing and QoL to subjectively feel this. The life motivation and purpose of life (questions 10 and 11) were also perceived through L2 acquisition by the majority of the respondents in the experimental group, but this was not visible in either of the control groups. The only drawback that was subjectively perceived as a slight issue in FLL by the experimental group was the fact that FLL required a lot of their time. This was confirmed by 19% of the respondents. However, on the whole, the results of the survey were very positive regarding the respondents´ increased wellbeing and furthermore improved QoL. When comparing the experimental group with both control groups, the results of the subjective feelings of wellbeing and happiness in the experimental group were overwhelmingly on the positive side.

## 4. Discussion

This section provides a wider context to the research that could help understand the differences in the experimental and control groups. As the results above illustrate, most of the participants of foreign language courses are females. The reason behind this is that generally women are more active in older age than men, and they outlive men by a few years in all European countries. The reasons for this discrepancy in terms of the statistical distribution of gender in society are as follows:

- Women more often participate in educational courses [35].
- Women’s life expectancy is higher [36].
- Women tend to be more sociable and therefore willing to take part in social events [35].
- Women tend to come in smaller groups or pairs [37].

These findings on women’s active participation in educational courses were also confirmed by Formosa and Findsen [38] in their study on the older adult population. In fact, they discovered that females outnumber males (3:1), with an increasing ratio when one concentrates purely on course attendance (5:1). 

In addition, the results reveal that three-quarters of the respondents from the experimental group lived in the cities/towns where there were more opportunities to take such language courses, as well as easier to access them, i.e., sufficient and frequent means of transport to the venue. Furthermore, 58% of those respondents possessed a university degree, which, as other research studies show, does not play a role in affecting the level of wellbeing [39]. 

As the findings in Table 1 demonstrate, older adults learn a foreign language to be able to make themselves understood while travelling abroad, enhance their cognitive skills, and extend their social networks—all these aspects improve their feelings of satisfaction and happiness. Similar results have also been found by Formosa [40], who stated that maintaining cognitive skills and expanding social contacts are the most common reasons for attending a U3A course. 

Thus, the findings of this study revealed that FLL positively contributed to the overall wellbeing of older people, being closely connected to the subjective positive feelings. This was expressed by their feelings of happiness, satisfaction of belonging to a group, and positive motivation to learn a foreign language. In addition, FLL positively affected their mental health and expanded their social networks, with this being subjectively perceived by the respondents. Therefore, generally, FLL is a meaningful activity that helps older people find their purpose of life, as well as providing life motivation, and this is also confirmed by the respondents. This active acknowledgment by the respondents themselves was very important as a motivator, and even if they knew that their learning outcomes were not very good, they still found FLL a major life motivator. 

These findings were supported by their answers to the open questions. Five respondents out of the total number of respondents admitted that FLL helps them increase their self-confidence. This has been also confirmed by Field [41], who stated that learning produces greater confidence and extends social networks. He also claimed that wellbeing is an important target for any intervention, and adult learning is an effective tool to enhance it. Furthermore, Narushima et al. [39] explained that participating in adult learning courses functions as a compensatory strategy for adults to help them develop psychological and social reserve capacities. Klimova and Pikhart [14] in their study showed that FLL courses generated novel opportunities for elderly people in the area of socializing and integration into society, which consequently may positively affect their wellbeing. Furthermore, their findings indicated that it is social and psychological well-being in particular through which the cognitive benefits of FLL might be observed. 

Steptoe et al. [42] have shown that wellbeing is directly connected to good health and longevity. Therefore, national governments and all stakeholders, such as health practitioners, social workers, gerontologists, and educators dealing with the present issue of aging population, should pay undivided attention to the enhancement of older people’s wellbeing by all possible intervention approaches, one of which is FLL. 

Young adult students, whose objective results in L2 vocabulary and grammar progress tests are generally very good, experienced almost the opposite to older learners due to the cognitive decline of the latter, and they did not reach very high levels of these positive feelings in comparison the experimental group. Younger students did not appear to feel motivated that much when learning a foreign language. We also rarely found very high levels of enjoyment from the learning process in comparison with the older generation, as can be seen in Table 2, i.e., the control group 1 results. However, they were aware of the fact that a foreign language will help them in their future career, as 93% of the respondents reported. This finding was also confirmed by Kormos and Csizer, who identified that the key motivator for FLL among university students was mainly to succeed in the job market [43].

There is usually a strong negative connection between the learning outcomes of older adult learners (that are very low, on the basis of the classic evaluation methods used after the course) and their learner’s satisfaction with the learning process per se. The reasons for this, as the questionnaire open question proved, are multifold, as follows:

- Enhanced social contacts;
- A new free-time activity;
- Meeting a younger generation (tutors of the classes);
- A new environment (university).

The negative learning outcomes of seniors are well documented in the scholarly literature [44], which describes the significant impact of aging on cognition. Even normal aging correlates with structural and functional cognitive changes, such as the decline of performance of cognitive tasks connected to working memory. Furthermore, there are changes in neuronal structure followed by the loss of synapses, which leads to dysfunction of neuronal networks [44]. The current research clearly shows the decline of crystallized (vocabulary) and fluid (processing speed) abilities [45]. The vocabulary knowledge seems to be rather stable with aging; however, only in the first language (L1) knowledge; in L2, new vocabulary acquisition shows a dramatic decline after the age of 60 years.

All these cognitive changes have a negative impact on short-, medium-, and long-term memory, and all of them are crucial for L2 acquisition. The decline of cognitive abilities is a natural process, but it does not deter seniors from being motivated to take up a new hobby-like activity, which any L2 acquisition is. Furthermore, seniors themselves admit difficulties with retaining new words as they are aware of the decline of their cognition [12], but they still reach very high levels of subjective satisfaction and motivation regardless of the language studied, their learning results, the age of the participant, etc. 

## 5. Conclusions

There is not much research comparing subjective positive feelings and the feelings of wellbeing between younger and older adults. These research findings could be very important for the development of neuroscience and cognitive science because it is important to specify to what extent the memory retention process deteriorates with age, as well as to determine the exact rate of this deterioration. These findings also seem very important for other social sciences, such as psychology and learning psychology. On the other hand, it is obvious that this deterioration does not mean corresponding dissatisfaction with the learning process itself. On the contrary, in these older learners, we can clearly observe an opposite trend, which leads to enhanced satisfaction and elevated feelings of enjoyment in the participants of the course. Moreover, as stated in the introduction, FLL might have a positive impact on the maintenance or even enhancement of seniors’ cognitive functions since brain plasticity persists even in older age [7,8,9,10,11,12,13,14].

This manuscript should initiate a further interest in the topic of FLL as a non-pharmacological approach to improved quality of life. Surprisingly, in younger adults, FLL does not work this way at all as a tool to improve their QoL, as revealed by this research, but in older adults, the wellbeing and subjective feelings of satisfaction results after their FLL activity are counterintuitive because their learning outcomes were shown to not reach very high levels but their satisfaction increased. Further research should test the initial state of the motivation and wellbeing in the participants and then compare it with the outcomes after the FLL course.

FLL can, thus, be another intervention method towards improved QoL and wellbeing of seniors along with other already well-accepted and documented methods such as active music performance and listening [46], various community activities [47], any lifelong learning that does not already include FLL [39], voluntary activities in/for the community [48], social (or even political) engagement [49,50,51], and civic engagement as such [52]. Positive psychology strategy [53] can be utilized to improve QoL and wellbeing of seniors who take up a new pursuit in their life, which an L2 acquisition is. To improve their QoL, L2 acquisition is a way that is manageable and relatively easy to attain.

The research was conducted with a relatively large group of respondents (*n* = 105) and two control groups of the same size as the experimental group; however, to double-check the results and make them more reliable, it would be helpful to conduct similar research on an international level. Despite the study’s national background and limitation (the Czech Republic only), we can still claim that the yielded results are very interesting and can be transferrable to an international context. 

Naturally, there are some reservations connected to the positive psychology perspective, claiming that it puts too much stress on positive aspects of life and suppresses the powerful impact of negative emotions and life situations on our wellbeing and life in general. There are even many conceptual and methodological limitations of positive psychology that aim at something that could be called positive psychology 2.0 [54]. The problem of the lack of a comprehensive theory of positive psychology is grave and creates a lot of misunderstanding, e.g., in our paper, we did not distinguish between subjective wellbeing and subjective positive feelings, which could be seen from the perspective of cognitive psychology as a lack of scientific rigor. Positive psychology 2.0 should not only focus on positive aspects and their impact on human life, but it should also implement the negative side of human life and study its influence when intertwined with positive life experience [54]. Further research could therefore be oriented this way, i.e., taking into account a traditional positive psychology perspective by adding the ideas of positive psychology 2.0, which could mean taking into consideration all aspects of human life, not only the positive ones. 

To sum up, the subjective feelings associated with L2 acquisition as a tool to improve the QoL of the elderly population was very high compared to the younger learners’ group, despite the fact that the learning outcomes in the latter group were considerably higher than in the group of seniors. Again, in this paper, we argued that the natural process of aging, with all its negative aspects connected to the deterioration and loss of memory, does not imply the deterioration of life satisfaction if supported by adequate tools, with FLL being one of them. Moreover, the acquisition of new skills or competencies, in this case L2 acquisition or FLL, can be an ideal intervention that could be utilized on a much larger scale to improve seniors´ QoL. 

## Figures and Tables

**Table 1:** An overview of the questionnaire results of the experimental group.

Question	Strongly Agree	Agree	Agree a Little	Disagree a Little	Disagree	Strongly Disagree
1. Learning a new language improves my concentration.	32.4%	52%	14.7%	0%	1%	0%
2. Learning a new language improves my memory.	40.2%	49%	7.8%	2%	1%	0%
3. Learning a new language improves my attention.	34.3%	42.2%	23.5%	0%	0%	0%
4. Learning a new language improves my health.	13.7%	43.1%	31.4%	6.9%	4.9%	13.7%
5. Learning a new language improves my creativity.	24%	50%	22%	1%	3%	0%
6. Learning a new language helps me find new friends.	35.3%	46.1%	16.7%	2%	0%	0%
7. Learning a new language helps me understand different cultures.	46.1%	42.2%	10.8%	0%	1%	0%
8. Learning a new language helps me while travelling.	62.4%	29.7%	5%	2%	1%	0%
9. Learning a new language helps me with learning other things, too.	25.7%	51.5%	18.8%	4%	0%	0%
10. Learning a new language helps me while looking for life motivation.	27.5%	42.2%	23.5%	3.9%	2.9%	0%
11. Learning a new language helps me with finding the purpose of my life.	14%	40%	28%	7%	9%	2%
12. Learning a new language is enjoyable.	42%	46.1%	7.8%	2%	2%	0%
13. Learning a new language brings me personal satisfaction.	37.3%	44.1%	15.7%	1%	0%	2%
14. Learning a foreign language brings me feelings of happiness.	37.3%	42.2%	15.7%	2.9%	1%	1%
15. Learning a foreign language is stressful.	2%	2%	21.8%	9.9%	41.6%	22.8%
16. Learning a new language does not bring any benefits to me.	0%	2%	6.9%	9.9%	44.6%	36.6%
17. Learning a new language can have a negative impact on me.	0%	1%	2%	3.9%	47.1%	46.1%
18. Learning a new language occupies a lot of my time.	2%	16.7%	25.5%	14.7%	30.4%	10.8%
19. Learning a new language is a positive motivation for me.	36.3%	46.1%	12.7%	2.9%	1%	1%
20. Learning a new language will be useful for me in the future.	36%	37%	19%	5%	3%	0%

**Table 2:** An overview of the questionnaire results of the young control group.

Question	Strongly Agree	Agree	Agree a Little	Disagree a Little	Disagree	Strongly Disagree
1. Learning a new language improves my concentration.	5%	25.7%	38.6%	6.9%	20.8%	3%
2. Learning a new language improves my memory.	12.7%	48%	26.5%	3.9%	7.8%	1%
3. Learning a new language improves my attention.	2%	20.6%	48%	8.8%	16.7%	3.9%
4. Learning a new language improves my health.	2.9%	5.9%	30.4%	15.7%	36.3%	8.8%
5. Learning a new language improves my creativity.	9.8%	39.2%	30.4%	4.8%	13.7%	2%
6. Learning a new language helps me find new friends.	22.5%	23.1%	17.6%	6.9%	7.8%	2%
7. Learning a new language helps me understand different cultures.	44.1%	38.2%	14.7%	1%	2%	0%
8. Learning a new language helps me while travelling.	80.4%	12.7%	2.9%	1%	2.8%	0%
9. Learning a new language helps me with learning other things, too.	22.5%	43.1%	20.6%	2.9%	7.8%	2.9%
10. Learning a new language helps me while looking for life motivation.	8.8%	18.6%	27.5%	10.8%	23.5%	10.8%
11. Learning a new language helps me with finding the purpose of my life.	4.9%	12.7%	25.5%	13.7%	27.5%	15.7%
12. Learning a new language is enjoyable.	16.7%	40.2%	28.4%	3.9%	9.8%	1%
13. Learning a new language brings me personal satisfaction.	4.9%	33.3%	31.4%	11.8%	15.7%	2.9%
14. Learning a foreign language brings me feelings of happiness.	7.8%	36.3%	28.7%	8.8%	16.7%	2%
15. Learning a foreign language is stressful.	2.8%	16.7%	20.6%	16.7%	38.2%	4.9%
16. Learning a new language does not bring any benefits to me.	0%	1%	3.9%	2%	46.1%	42.1%
17. Learning a new language can have a negative impact on me.	0%	2.9%	10.8%	6.9%	51%	28.4%
18. Learning a new language occupies a lot of my time.	2.9%	28.4%	32.7%	24.5%	9.8%	2%
19. Learning a new language is a positive motivation for me.	4.9%	34.3%	29.2%	7.8%	10.8%	2.9%
20. Learning a new language will be useful for me in the future.	61.8%	31.4%	5.9%	1%	0%	0%

**Table 3:** An overview of the questionnaire results of the elderly control group.

Question	Strongly Agree	Agree	Agree a Little	Disagree a Little	Disagree	Strongly Disagree
1. My hobbies improve my concentration.	0%	23.7%	44.7%	13.2%	15.8%	2.6%
2. My hobbies improve my memory.	0%	28.9%	23.7%	2.6%	34.2%	10.5%
3. My hobbies improve my attention.	0%	23.7%	36.8%	10.5%	21.1%	7.9%
4 My hobbies improve my health.	5.3%	26.3%	26.3%	5.3%	18.4%	18.4%
5. My hobbies improve my creativity.	0%	18.4%	31.6%	10.5%	26.3%	13.2%
6. My hobbies help me find new friends.	2.6%	18.4%	18.4%	0%	23.7%	36.8%
7. My hobbies help me understand different cultures.	2.6%	10.5%	15.8%	5.3%	28.9%	36.8%
8. My hobbies help me while travelling.	2.6%	10.5%	15.8%	2.6%	28.9%	39.5%
9. My hobbies help me with learning other things, too.	0%	13.2%	26.3%	5.3%	28.9%	26.3%
10. My hobbies help me while looking for life motivation.	5.3%	21.1%	50%	7.9%	13.2%	2.6%
11. My hobbies help me with finding the purpose of my life.	2.7%	18.9%	40.5%	16.2%	18.9%	2.7%
12. My hobbies are enjoyable.	23.7%	73.7%	2.6%	0%	0%	0%
13. My hobbies bring me personal satisfaction.	7.9%	81.6%	7.9%	0%	2.6%	0%
14. My hobbies bring me feelings of happiness.	7.9%	55.3%	34.2%	0%	2.6%	0%
15. My hobbies are stressful.	0%	0%	5.3%	5.3%	73.7%	15.8%
16. My hobbies do not bring any benefits to me.	0%	13.2%	7.9%	31.6%	36.8%	10.5%
17. My hobbies can have a negative impact on me.	10.5%	10.5%	21.1%	10.5%	34.2%	13.2%
18. My hobbies occupy a lot of my time.	2.6%	34.2%	36.8%	10.5%	13.2%	2.6%
19. My hobbies bring a positive motivation for me.	2.6%	50%	31.6%	7.9%	5.3%	2.6%
20. My hobbies will be useful for me in the future.	0%	28.9%	34.2%	7.9%	21.1%	7.9%
